# Is there a Role for Cyclophilin Inhibitors in the Management of Primary Biliary Cirrhosis?

**DOI:** 10.3390/v5020423

**Published:** 2013-01-24

**Authors:** Shawn T. Wasilenko, Aldo J. Montano-Loza, Andrew L. Mason

**Affiliations:** Department of Medicine, Zeidler Ledcor Centre, University of Alberta Hospital, Edmonton, Alberta, Canada; E-Mails: shawnw@ualberta.ca (S.W.), montanol@ualberta.ca (A.M.-L.)

**Keywords:** primary biliary cirrhosis, de novo autoimmune hepatitis, cyclosporine A, viral pathogenesis

## Abstract

Autoimmune hepatitis (AIH) and primary biliary cirrhosis (PBC) are poorly understood autoimmune liver diseases. Immunosuppression is used to treat AIH and ursodeoxycholic acid is used to slow the progression of PBC. Nevertheless, a proportion of patients with both disorders progress to liver failure. Following liver transplantation, up to a third of patients with PBC experience recurrent disease. Moreover a syndrome referred to as “*de novo* AIH” occurs in a proportion of patients regardless of maintenance immunosuppression, who have been transplanted for disorders unrelated to AIH. Of note, the use of cyclosporine A appears to protect against the development of recurrent PBC and *de novo* AIH even though it is a less potent immunosuppressive compared to tacrolimus. The reason why cyclosporine A is protective has not been determined. However, a virus resembling mouse mammary tumor virus (MMTV) has been characterized in patients with PBC and AIH. Accordingly, we hypothesized that the protective effect of cyclosporine A in liver transplant recipients may be mediated by the antiviral activity of this cyclophilin inhibitor. Treatment of the MMTV producing MM5MT cells with different antivirals and immunosuppressive agents showed that both cyclosporine A and the analogue NIM811 inhibited MMTV production from the producer cells. Herein, we discuss the evidence supporting the role of MMTV-like human betaretrovirus in the development of PBC and *de novo* AIH and speculate on the possibility that the agent may be associated with disease following transplantation. We also review the mechanisms of how both cyclosporine A and NIM811 may inhibit betaretrovirus production *in vitro*.

## 1. Primary Biliary Cirrhosis

Primary biliary cirrhosis (PBC) is rare autoimmune liver disease that predominantly affects women. It is characterized by immune destruction of intrahepatic bile ducts, the aberrant expression of mitochondrial antigens on biliary epithelium and the development of anti-mitochondrial antibodies (AMA) [1]. The progressive loss of bile ducts leads to accumulation of bile in the liver, fibrosis and then cirrhosis in those unresponsive to therapy. The only licensed treatment for PBC is ursodeoxycholic acid (UDCA) therapy, which acts as a choleretic agent to eliminate bile from the liver [2,3]. However, a third of patients still develop progressive disease and as a result, PBC accounts for 10% of patients requiring liver transplantation in developed countries [4].

Apart from the study of UDCA, clinical trials for PBC have been mainly geared towards investigating immunosuppressive agents. This is because immunosuppression has proven life saving for patients with autoimmune hepatitis (AIH). However, the outcomes of similar clinical studies in PBC have been disappointing [5]. Individual treatments have had little impact on halting the progression of PBC and specific immunosuppressive agents have not therefore, been adopted because of toxicity or lack of efficacy [6].

Of interest, cyclosporine A (CsA) is one of the few drugs that has shown some evidence of reducing liver related mortality [7]. However, CsA is not used in non-transplant patients due to adverse effects on renal function and blood pressure. Even though immunosuppression has little role in the management of PBC, an overlap syndrome of PBC and AIH is recognized to occur where patients manifest with features of both diseases and immunosuppression may be of value in disease management [1,5,8]. While the biochemical hepatitis component of this disorder can be controlled somewhat with corticosteroid therapy, the effect has not been systematically studied in controlled trials [1].

The treatment of choice for patients with liver failure secondary to PBC is liver transplantation. Even though the disease may reoccur, the outcomes are usually favorable [9]. More than two thirds of patients develop both AMA and the PBC specific phenotype in bile ducts with aberrant expression of mitochondrial autoantigens—the “mitochondrial phenotype”. However, only a proportion of patients develop biochemical and histological disease. The frequency of recurrent PBC usually increases progressively with time and histological disease is reported in about 30% of patients after 10 years of the liver transplant [9,10,11,12,13,14,15,16,17,18].

**Figure 1:**
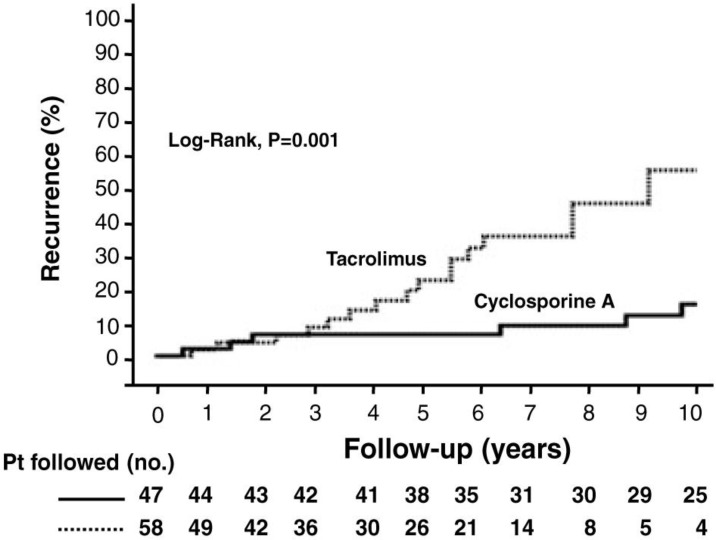
**Cumulative probability of primary biliary cirrhosis recurrence after liver transplantation according to use of cyclosporine A (—) and tacrolimus (- – -).** The 5-year probability of recurrence was 7% and 21%, respectively (p = 0.001, log-rank test). The 10-year probability of recurrence was 13% and 58% in these same groups, but fewer patients were followed. (With permission of John Wiley and American Journal of Transplantation).

Another consistent observation from many transplant programs is that the more potent immunosuppressive regimens using tacrolimus accelerates the onset and severity of recurrent disease. In contrast, CsA based regimens have been linked to a diminished incidence of recurrent disease [11,16]. For example, we found that the probability of recurrent PBC at 10 years was 58% in patients on tacrolimus as compared to 13% of patients on CsA from a cohort of more than a thousand transplant recipients (Figure 1) [13]. We also found that primary immunosuppression with CsA as compared to tacrolimus was associated with eight-fold reduction in the risk of PBC recurrence, in agreement with other liver transplant centers [11,13,19].

The immunosuppression regimens used in the 1990s probably contributed to an era effect, when PBC patients undergoing liver transplantation experienced a lower incidence of disease recurrence. Others liver transplant centers have reported a similar protective era effect that has been linked with several factors including specific immunosuppression regimens, the use of younger donors and decreased cold ischemic time [11,16]. Nevertheless, a major conclusion of most series documenting outcomes in patients with PBC following liver transplantation is that CsA is associated with a lower incidence of recurrent disease [10,11,12,13,14,15,16,18]. Also, of interest, the primary use of CsA has been shown to confer a protective effect against other autoimmune diseases, such as *de novo* AIH in liver transplant recipients in general [20] as well as a decreased risk of recurrent or *de novo* inflammatory bowel disease [21] after liver transplantation for primary sclerosing cholangitis (PSC).

## 2. *De Novo* Autoimmune Hepatitis

AIH is a heterogeneous disorder observed in pediatric and adult populations with a variable presentation and prognosis. The diagnosis is usually established by an exclusion of other causes of liver disease, liver histology and the presence of a variety of autoantibodies. Also included in the spectrum of AIH are the poorly understood overlap syndromes with both PBC and PSC; however, the overlap syndromes are considered contentious because both PBC and PSC are exclusion criteria for making a diagnosis of AIH [1,5,8]. *De novo* AIH has been recognized for over a decade as a condition that affects patients transplanted for hepatic disorders other than AIH [22,23,24,25,26,27,28,29,30,31,32]. Comparable to classical AIH, the diagnosis of *de novo* AIH is essentially based on the presence of autoantibodies, distinctive histological findings and the exclusion of other conditions, such as viral hepatitis, acute or chronic rejection and immune mediated biliary disease [33]. Similar to the diagnosis of AIH in the general population, it has to be acknowledged that the diagnosis of *de novo* AIH following liver transplantation is not clear-cut.

While we lack evidence-based diagnostic criteria to distinguish the differing entities, there are strong similarities between *de novo* AIH, the recurrence of AIH in liver transplant recipients, and the classical AIH in the non-transplant setting. In a study based at our center we found that the probability of *de novo* AIH was approximately 4% at 10 years with an overall incidence of 4 cases per 1000 patient-years. It is notable that the frequency of *de novo* AIH may be higher than the prevalence of AIH in the general population, probably because transplant patients are exposed to more potential risk factors [20].

**Figure 2:**
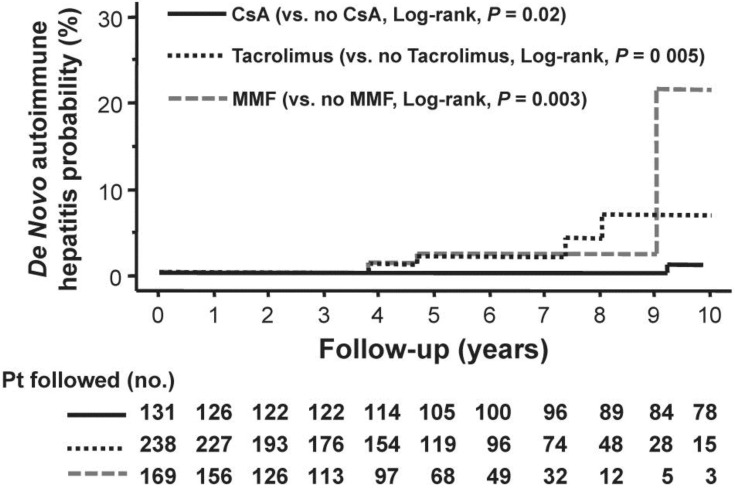
**Cumulative probability of *de novo* autoimmune hepatitis according to the use of cyclosporine A (—), tacrolimus (^…..^) and mycophenolate mofetil (- - -).** The 5- and 10-year probability of development of *de novo* autoimmune hepatitis with cyclosporine A was 0 and 1.2% respectively; the 5- and 10-year probability of development with tacrolimus was 1.9% and 6.0% respectively; and the 5- and 10-year probability of development with mycophenolate mofetil was 3.1 and 22.5% respectively. (With permission of John Wiley and Sons and Liver International).

With regard to immunosuppression usage, liver transplant recipients maintained on CsA had a 4-fold lower risk of *de novo* AIH, whereas those receiving tacrolimus or mycophenolate mofetil had a 4- and 6-fold higher risk of *de novo* AIH, respectively (Figure 2) [20]. Intriguingly, we found that patients who had donors aged 40 years or older or female donors had a 7-fold and 3-fold higher risk of developing *de novo* AIH, respectively. Moreover, female recipients with gender mismatch were protected against *de novo* AIH, reducing the risk by 10-fold [20]. In other words, having a younger male donor and primary use of CsA is protective against the development of *de novo* AIH.

Similar to patients with PBC, the immunosuppression regimens used in the 1990s probably contributed to the cohort effect observed in this study. Patients undergoing liver transplantation in this period experienced a 12-fold lower risk of *de novo* AIH compared with patients transplanted in the decade following 2000. It can be argued that the protective effect of CsA could also be attributable to the concomitant use of steroids, as previous studies have shown a role for steroid use in preventing development of *de novo* AIH [31].

## 3. Pathophysiology of Recurrent PBC and *de novo* AIH

While the genetic and environmental factors that trigger PBC are poorly understood, one could argue that the same mechanisms likely mediate recurrent disease in the allograft. There is a strong heritable component to disease that is difficult to quantify. The risk of developing PBC in family members ranges from 1% to 7% and PBC is more prevalent in monozygotic as compared to dizygotic twins [34,35]. Several case control and genome wide association studies have linked PBC with the HLA DR and DQ class II region, the IL-12/interferon γ cytokine axis and other genes associated with host pathogen interaction [36,37,38,39,40,41,42]. Collectively, the susceptibility data suggest a hypothesis that patients with PBC have a global disturbance in sensing and response to environmental agents [37]. It is tempting to speculate, therefore, that the penetrance of recurrent disease maybe related in part to the acquisition of protective alleles in the new allograft that prevents establishment of an infectious process but data are lacking to support this conjecture.

The recurrence of PBC following transplantation strongly suggests an infectious process and epidemiological data are suggestive of a transmissible etiology as well. Spouses, other unrelated family members, and even care providers have been reported to develop PBC suggesting a role for environmental factors in disease [43,44]. Also, PBC has been reported to cluster in specific geographical regions [44,45] and migration studies show that the children develop the relative incidence of their adopted host country, whereas their parents do not [46,47]. With regard to environmental agents, xenobiotics, specific bacteria and a betaretrovirus have been linked with the development of PBC, but none has been confirmed to cause the disease to date [48].

Our group first characterized a human betaretrovirus resembling the mouse mammary tumor virus (MMTV) in patients with PBC nearly ten years ago [49,50]. The initial finding was important because the betaretrovirus was shown to trigger the mitochondrial phenotype of PBC *in vitro* and was found in cells displaying the mitochondrial phenotype *in vivo* [50]. While 75% of patients with PBC had evidence of the HBRV in their peri-hepatic lymph nodes, the virus was difficult to detect in the liver and questions were raised concerning the validity of the original findings [51]. Accordingly, studies are ongoing to demonstrate proviral integrations in patients with liver disease to provide more substantial evidence that HBRV is a potential human pathogen [48].

It is understood that multiple layers of proof will be required to link HBRV with the pathogenesis of PBC and there are several inter-related ongoing studies that aim to address this issue. On and above demonstrating proviral integration in the bile ducts of patients with PBC, an ELISA is being constructed to determine the seroprevalence of HBRV infection in a large cohort of patients with liver disease. Also, mouse models are being used to demonstrate how infection with the closely related MMTV triggers autoimmune biliary disease [52]. These animal models are being used to validate combination antiviral treatments to inhibit betaretrovirus infection and ongoing clinical trials are investigating whether anti-retroviral therapy can improve histological, biochemical and clinical endpoints in patients with PBC [6,53,54].

Accordingly, we are working on the model that a betaretrovirus may trigger viral cholangitis in susceptible individuals with specific HLA DR and DQ class II alleles and genetic polymorphisms associated with the IL-12 cytokine axis. A similar model could be used to propose that factors that trigger the original disease also persist in the allograft. While more than two thirds of patients develop both AMA and the mitochondrial phenotype in bile ducts, only one third of patients develop biochemical and histological disease recurrence 10 years following transplantation. These data suggest that approximately one third of the patients with PBC who receive a liver transplant are protected against recurrent disease. The evidence supports the hypothesis that the environmental trigger(s) may persist in about two thirds of patients, whereas only a third develop progressive disease. Whether the factors modulating disease recurrence are protective polymorphisms such as those involving the HLA or IL-12 alleles in the allograft or the use of younger donors with shorter cold ischemic times remains to be resolved. However, a major factor that has reproducibly been shown to be protective at this juncture is the primary use of CsA.

Little is known about the etiology and pathogenesis of AIH in the non-transplant setting and the development of *de novo* AIH remains a total mystery. Indeed, three diverse processes could be involved with the pathogenesis of *de novo* AIH including autoimmunity, a *forme fruste* of allograft rejection and the possibility of a viral infection. In fact, it has been suggested that AIH may represent serologically unidentified viral infection(s) [5] and we have found evidence of HBRV infection in patients with AIH. In one case with acute onset AIH, the viral load was highest with acute presentation and diminished by corticosteroid treatment [55]. This observation lead to the proposal of a model that the use of corticosteroids may have the dual purpose of limiting viral spread and diminishing inflammation [55]. Indeed the related agent, MMTV, has an obligate prerequisite for replicating in dividing lymphocytes. In the setting of liver transplantation, this model would also be consistent with the antiviral and anti-lymphocytic activity of CsA in protecting against the development of *de novo* AIH.

## 4. Is There a Potential Role of Cyclophilin Inhibitors in PBC?

The observation that the more potent immunosuppressant, tacrolimus is associated with earlier and more severe recurrence of PBC suggests two potentially compatible hypotheses. The first is that tacrolimus inhibits the immune system to a greater degree permitting the emergence of an infectious agent. The second is that CsA may be acting in part as an antiviral in patients with PBC following liver transplantation. It has to be acknowledged, however, that data demonstrating recurrent HBRV in the allograft are lacking to support these hypotheses. Nevertheless, we have found that CsA and the analogue NIM811 can inhibit betaretrovirus production suggesting a critical role for cyclophilins in the replication and infectivity of betaretroviruses [19].

**Figure 3:**
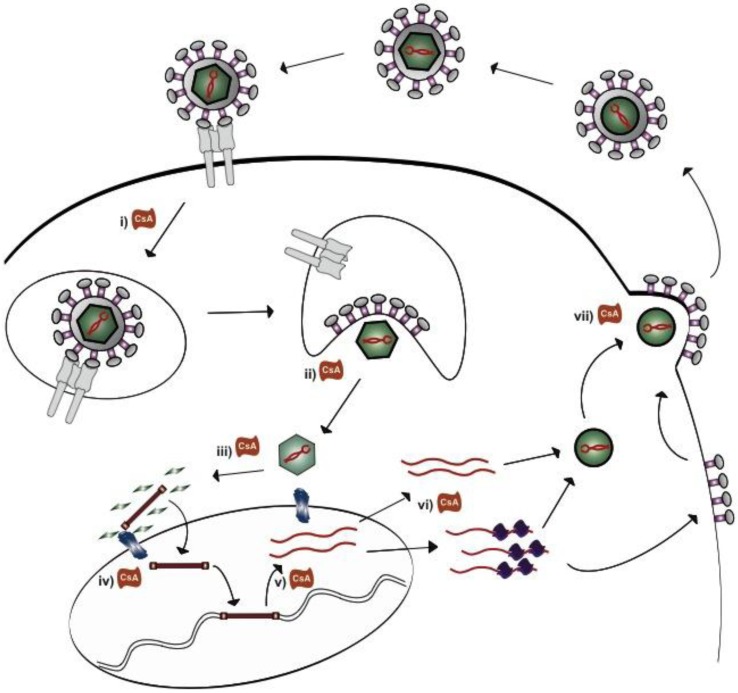
**Cyclophillin inhibitors may block the betaretrovirus life cycle at distinct steps.** The early events of retroviral infection include (**i**) internalization following receptor engagement, (**ii**) disassembly and (**iii**) uncoating during reverse transcription, (**iv**) nuclear import of the preintegration complex and proviral integration. In the MMTV producing Mm5MT cells, antiviral blockade with cyclophilins can impact on the more downstream events such as (**v**) RNA transcription, (**vi**) pre-translational and post-translational protein processing as well as (**vii**) viral budding and maturation.

For our *in vitro* studies, we used the MM5MT mouse breast cancer cells containing integrated MMTV provirus for testing the sensitivity of antiviral and immunosuppressive agents to diminish MMTV production. By design, this model cannot be used to test early events in the retrovirus life cycle, such as viral internalization, uncoating, disassembly, reverse transcription, nuclear import of the preintegration complex and proviral integration (Figure 3). Nevertheless, the downstream production of infectious particles from integrated provirus can be studied.

In these *in vitro* studies, we observed a reduction in reverse transcriptase activity and viral genome levels in supernatants from MM5MT cells incubated with the cyclophilin inhibitors, CsA and NIM811, whereas tacrolimus and reverse transcriptase inhibitors had significantly less effect [19]. Steady state viral protein levels were unaffected by the presence of CsA or NIM811, however. We observed change in protease processing of MMTV Gag proteins with either CsA or NIM811, whereas the combination HIV protease inhibitor, lopinavir and ritonavir (Kaletra^TM^) partially inhibited the production of the MMTV p27 Capsid protein [19]. It is clear however that the likely block in viral replication is independent of the immunosuppressive nature of CsA as incubation with NIM811 resulted in similar levels of reverse transcriptase and genome equivalents. At this point however, the mechanism(s) of how either CsA or NIM811 block viral production has yet to be resolved.

## 5. How do Cyclosporine A and NIM811 Decrease Betaretrovirus Production

Reviewing the betaretroviral life cycle helps us to understand the critical steps that CsA and NIM811 may interfere with viral replication (Figure 3). As discussed, the use of Mm5MT cells with a stably integrated MMTV provirus prevented the study the early events of infection. Nevertheless, cyclophilin antagonists may function to prevent internalization of virions as observed with several agents including human papilloma virus (HPV), measles virus and HIV. For example, both CsA and NIM811 counteract cyclophilin B enabling the necessary conformational changes of the HPV minor capsid protein required for viral internalization [56,57]. It is also known that both cyclophilin A and cyclophilin B assist the cellular entry of HIV and measles virus, respectively, through a critical interaction with CD147, a transmembrane glycoprotein belonging to the immunoglobulin superfamily [58,59]. However, no one has demonstrated to date that CsA or its analogues specifically function by blocking CD147 mediated viral entry.

Cyclophilins also play an important role in viral uncoating and disassembly. For HPV this process occurs in endosomal compartments with the assistance of cyclophilin B, for example, and this activity inhibited by CsA [57]. Another example of cyclophilin mediated viral replication includes both the disassembly of mature cores and the nuclear import of the HIV preintegration complex (Figure 3iii). Cyclophilins play a critical role in assisting the interaction of the viral capsid protein with the major cytoplasmic component of the nuclear pore complex, NUP358 necessary for uncoating the capsid and shuttling of the preintegration complex into the nucleus [60,61,62]. In this case, inhibition of the cyclophilin A interaction with HIV capsid by CsA impairs the passage of the mature viral core to the nuclear pore complex.

Following proviral integration, cyclophilins have been shown to play a role in several processes in the viral life cycle comprising viral RNA and protein production, assembly and maturation (Figure 3). For example, CsA has been shown to block the κB activation of the HIV enhancer region to inhibit viral RNA transcription [63]. Other agents, such as hepatitis C virus (HCV) rely on cyclophilin A interactions with viral components of the replication complex to produce RNA transcripts, a process also blocked by CsA [64,65,66,67,68,69,70,71]. In a similar vein, CsA blocks coronavirus replication via an unknown mechanism at a step prior to RNA and protein synthesis [72]. While there is significant anecdotal evidence to favor the inhibition of RNA transcription, the presence of CsA and tacrolimus can potentiate, rather than block, transcriptional activity of the MMTV promoter [73]. However, this process is modulated by dexamethasone activation of the glucocorticoid responsive elements in the long terminal repeat.

It is also possible cyclophilin inhibitors negatively regulate viral protein steady state levels at any one of the many steps involved in viral protein production and turnover. For example, CsA blocks nuclear export of influenza RNA transcripts [74,75,76]. Furthermore, processing of the HCV polyprotein into functional components requires the support of cyclophilin A interacting with NS2 that can be blocked by CsA and Debio-025 [77,78,79]. The presence of CsA also accelerates proteolytic degradation of the influenza matrix protein M1 by augmenting its interaction with Cyclophilin A. In contrast, cyclophilin inhibitors trigger loss of the HIV Vpr protein—a known partner of cyclophilin A—independent of proteosome activity [80].

As discussed for our *in vitro* experiments with MMTV producing cells, it is unlikely that the cyclophilin inhibitors impact on any of the steps from viral entry to viral transcription and protein production given that CsA and NIM811 treatment did not impact on MMTV RNA or protein levels in cells [19,73]. These data suggest that viral transcription and protein production likely proceeds appropriately in producer cells. Therefore, additional studies should be directed towards investigating viral assembly, maturation and the infective stages of the viral life cycle as numerous viruses utilize cyclophilins during target cell infection. Specifically, we know that HIV incorporates cyclophilins into budding virions and interference of this process by CsA or NIM811 reduces infectivity [61,81,82]. These data suggest a model that virion incorporated cyclophilin, or target cell cyclophilin may be necessary for effective internalization, uncoating and/or nuclear import of the MMTV genome.

## 6. Prospectus

At present, patients with PBC have limited options for therapeutic intervention. Clearly, better treatments are required for the ~ 20% of patients that progress to liver failure and to treat those with recurrent disease following liver transplantation. While anti-retroviral therapy for PBC has shown promise with improvements in hepatic biochemistry and histology [6,53,54,83], the specific drugs employed in clinical trials to date have been manufactured for treating HIV rather than HBRV. Our preliminary data support the hypothesis that CsA and other cyclophilin inhibitors may inhibit betaretrovirus activity during maturation or infective stages of target cells. As such, further characterization of the role of cyclophilins in the betaretrovirus life cycle would be beneficial both *in vitro* and in mouse models of PBC with MMTV infection. [52] Such studies are encouraged as they would serve the dual purpose of characterizing the mechanism of cyclophilin inhibitors in blocking HBRV production and for identifying novel management strategies for patients with PBC.
